# Supplementary figures and images for: Automatic wound detection and size estimation using deep learning algorithms
Source: PLoS Comput Biol. 2022 Mar 11;18(3):e1009852. doi: 10.1371/journal.pcbi.1009852 (PMC8942216; doi:10.1371/journal.pcbi.1009852)

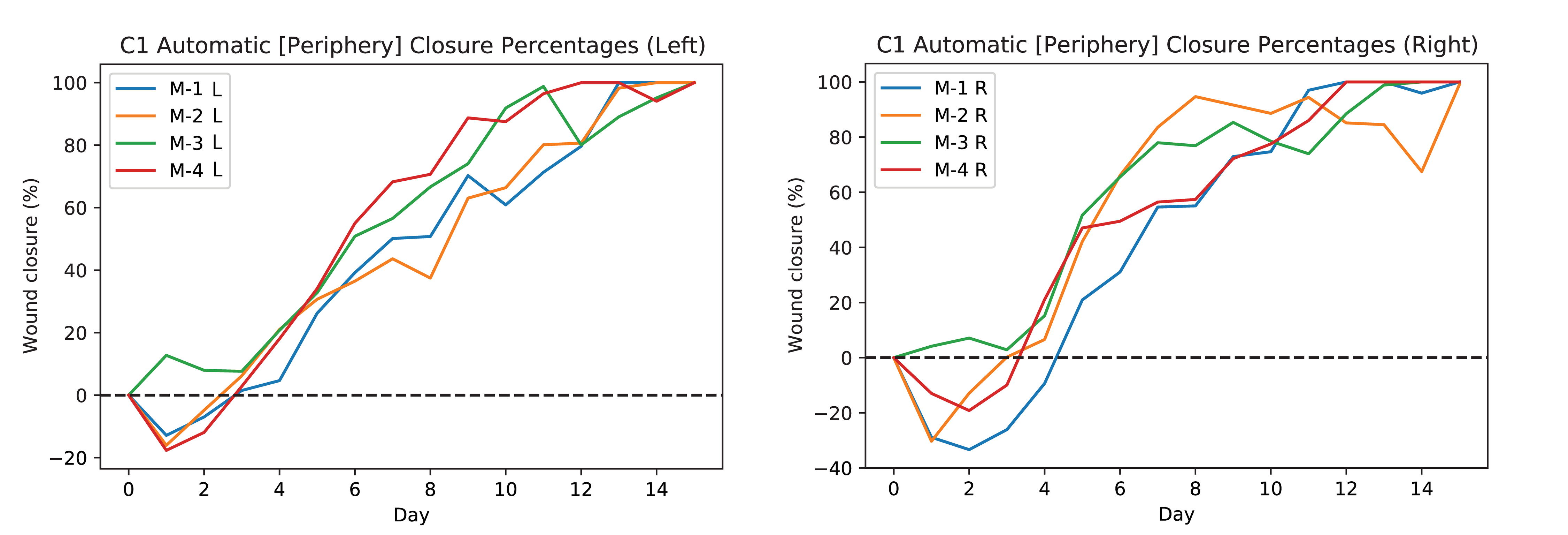

Supplement: S1 Fig — (TIF) [file pcbi.1009852.s001.tif]

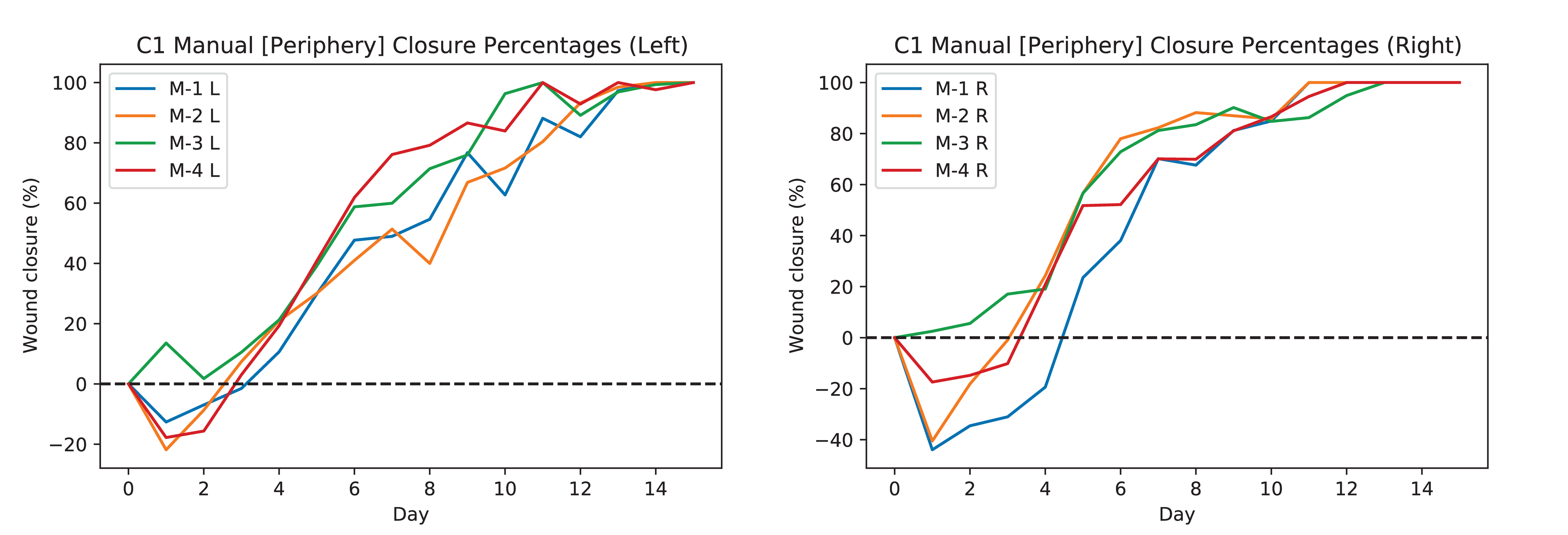

Supplement: S2 Fig — (TIF) [file pcbi.1009852.s002.tif]

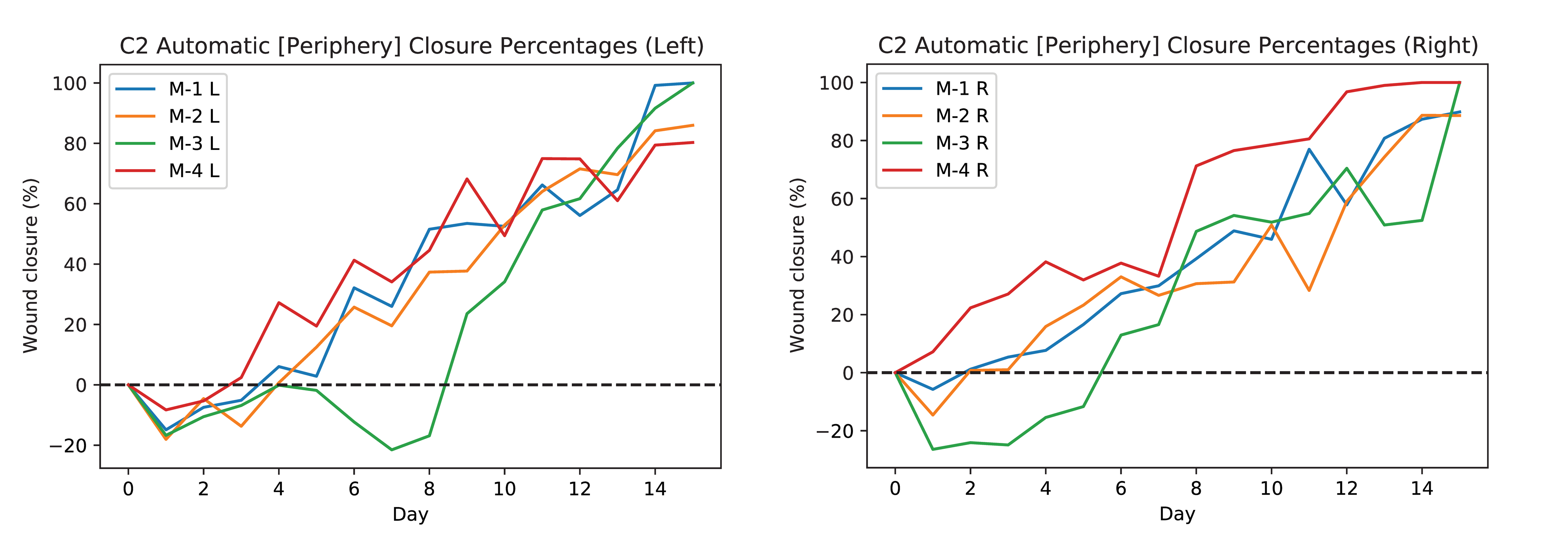

Supplement: S3 Fig — (TIF) [file pcbi.1009852.s003.tif]

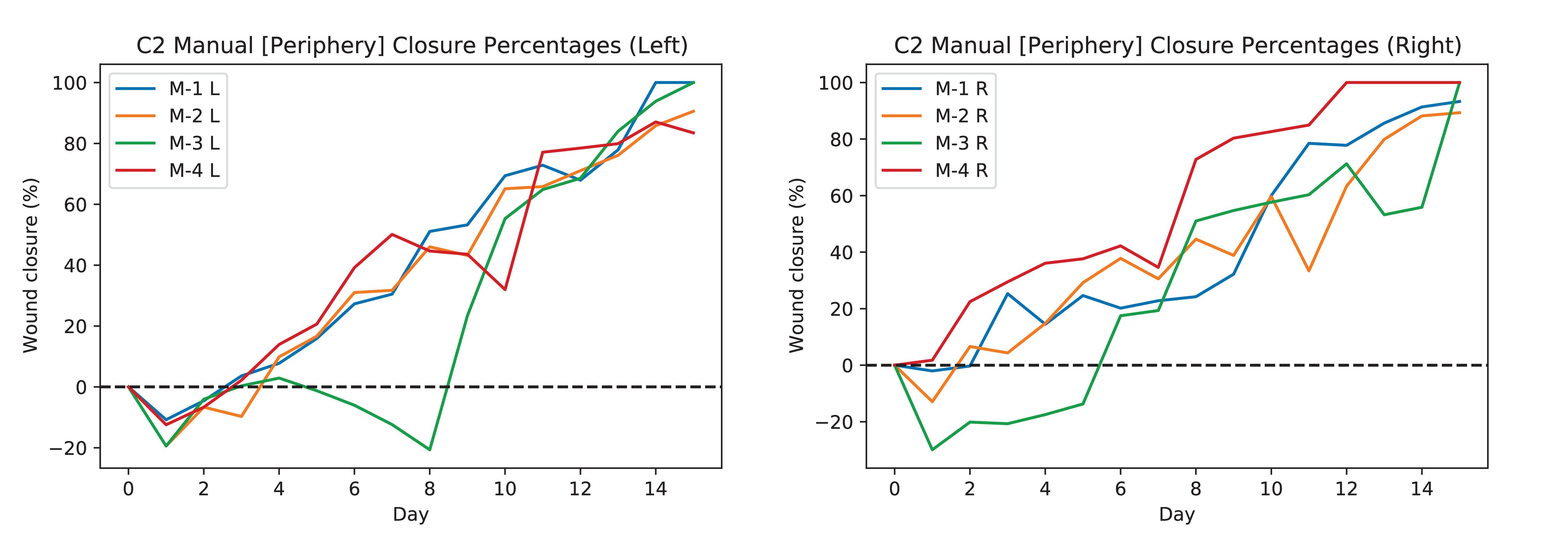

Supplement: S4 Fig — (TIF) [file pcbi.1009852.s004.tif]

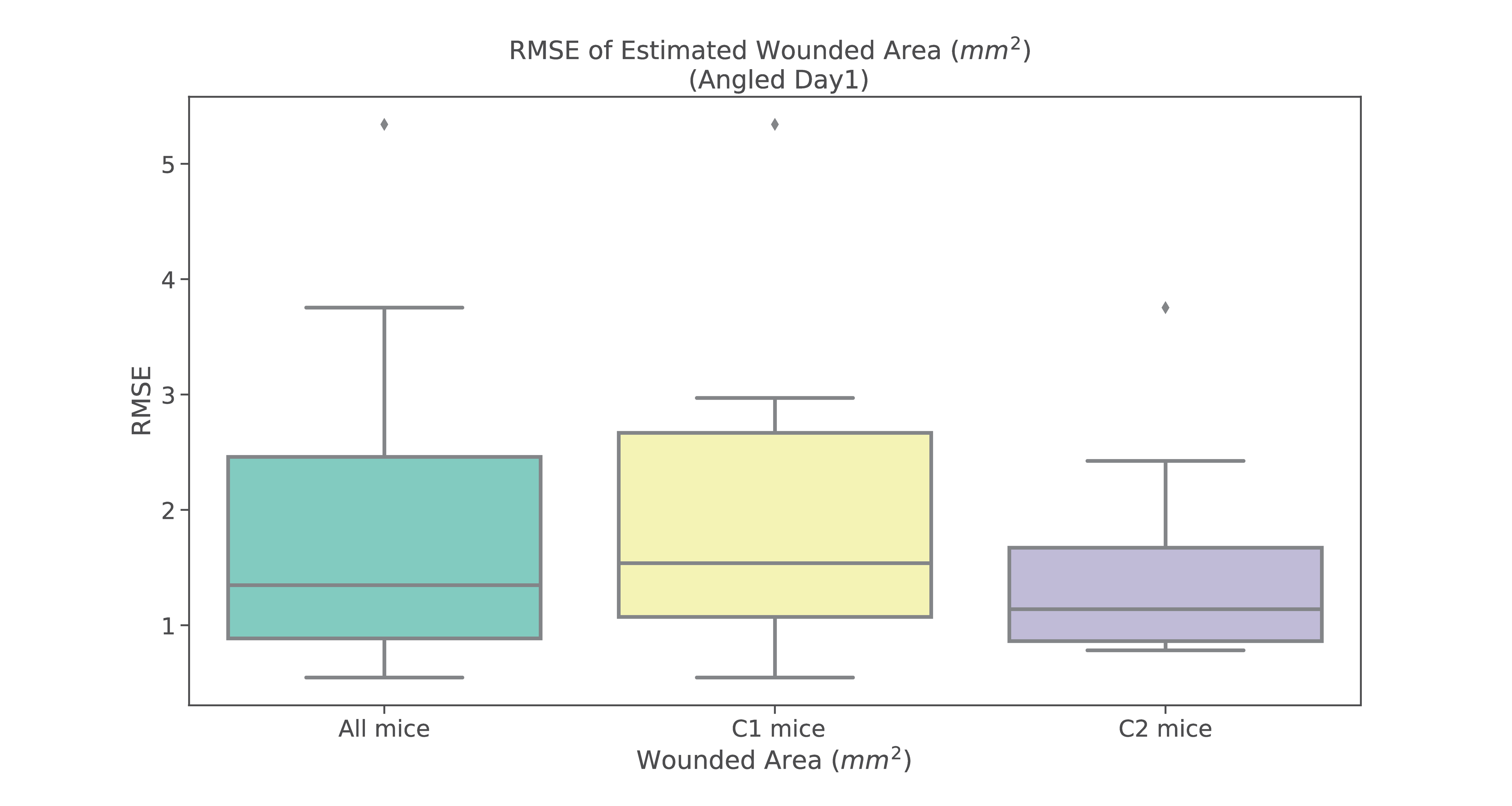

Supplement: S5 Fig — (TIF) [file pcbi.1009852.s005.tif]

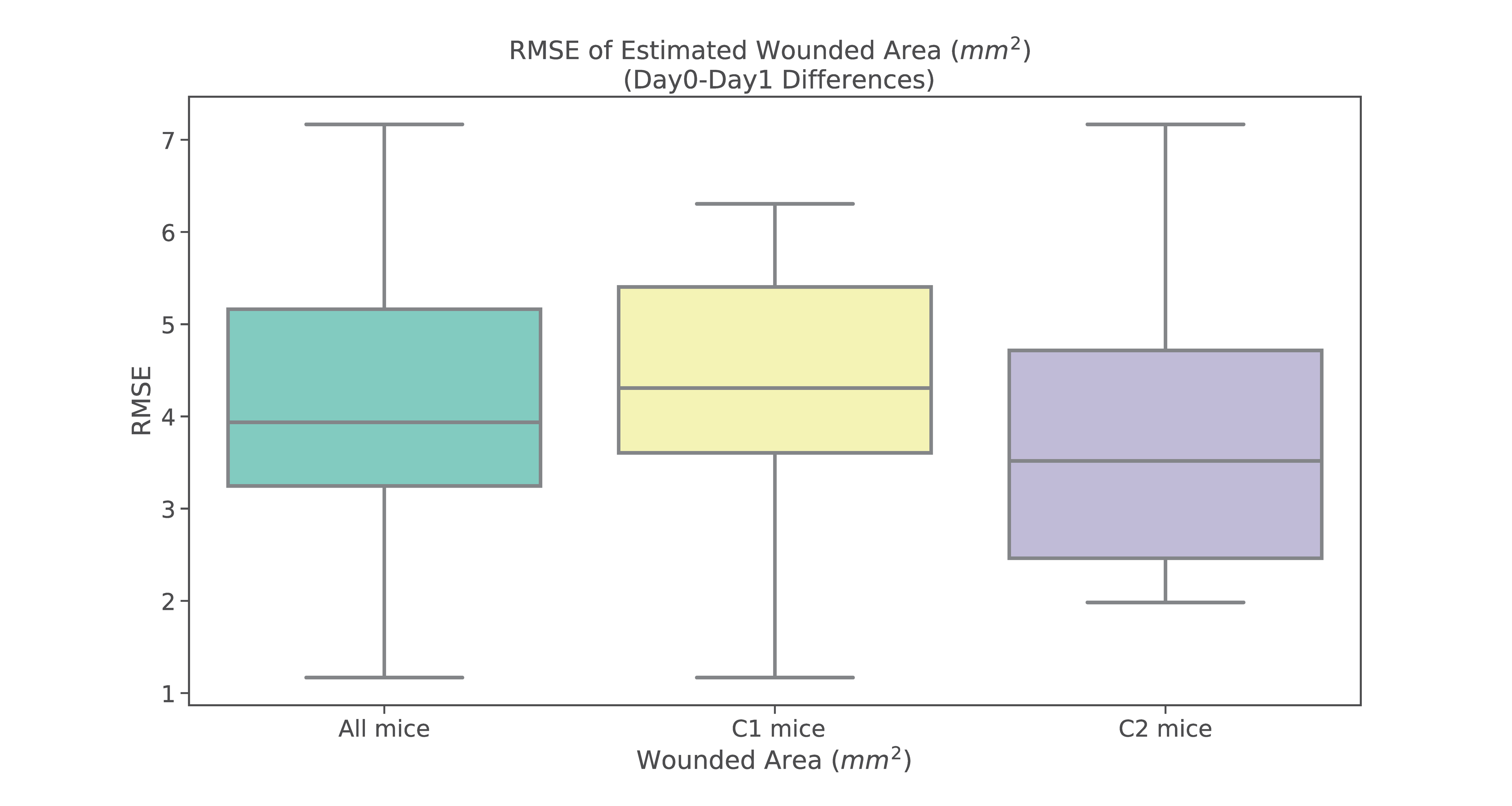

Supplement: S6 Fig — (TIF) [file pcbi.1009852.s006.tif]

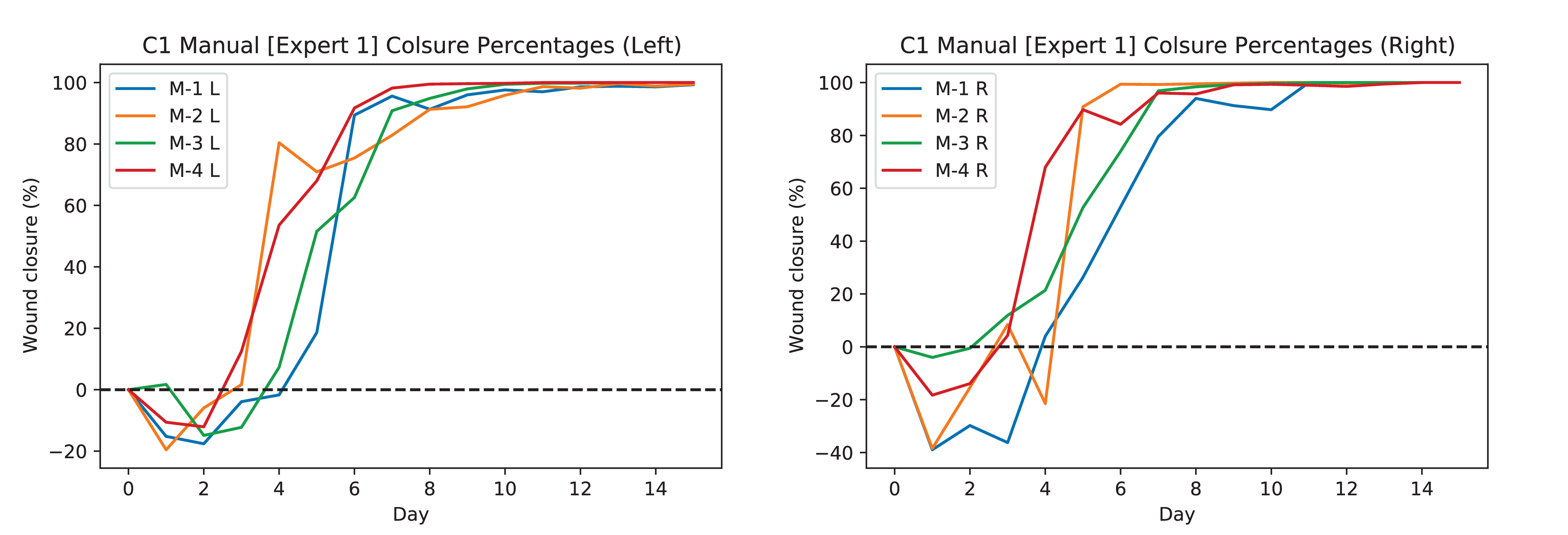

Supplement: S7 Fig — (TIF) [file pcbi.1009852.s007.tif]

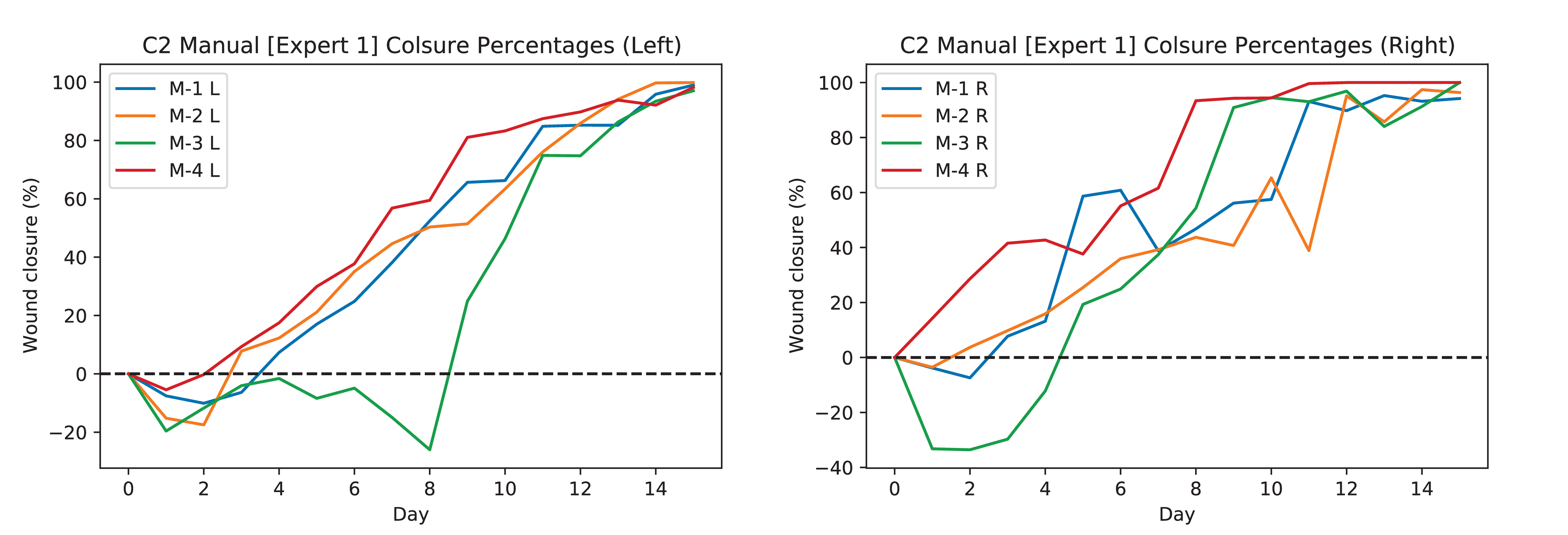

Supplement: S8 Fig — (TIF) [file pcbi.1009852.s008.tif]

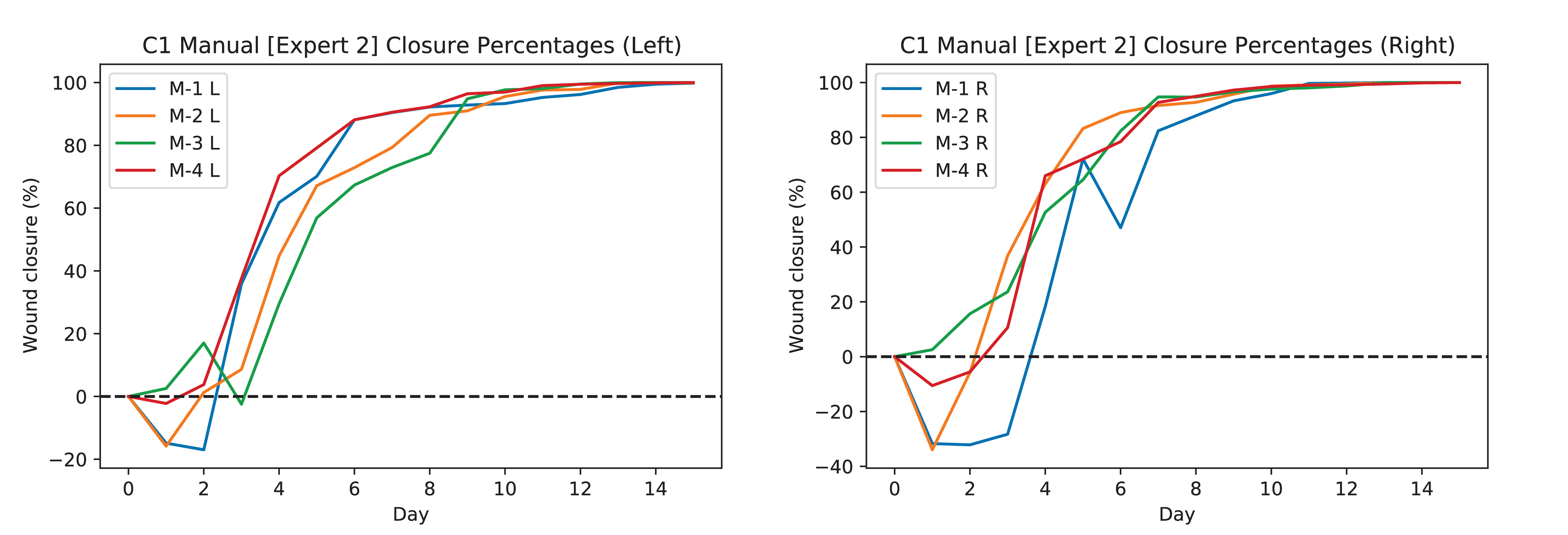

Supplement: S9 Fig — (TIF) [file pcbi.1009852.s009.tif]

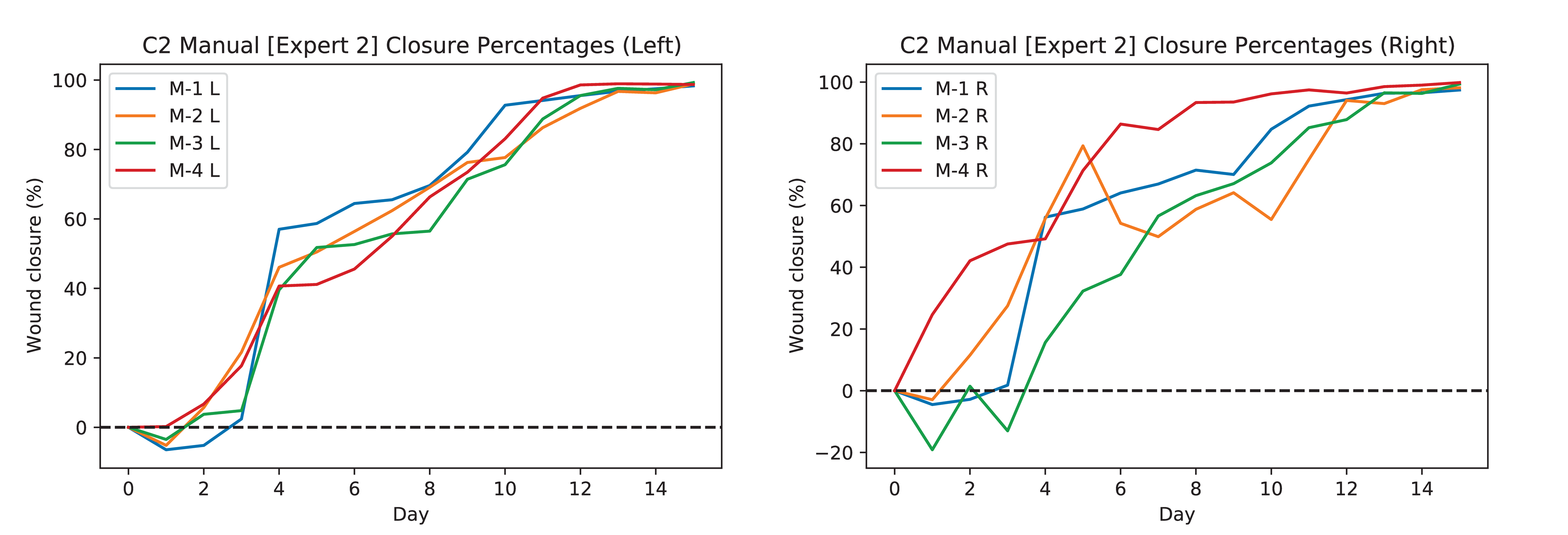

Supplement: S10 Fig — (TIF) [file pcbi.1009852.s010.tif]
